# Supplementary figures and images for: Prognostic values of the core components of the mammalian circadian clock in prostate cancer
Source: PeerJ. 2021 Dec 9;9:e12539. doi: 10.7717/peerj.12539 (PMC8667750; doi:10.7717/peerj.12539)

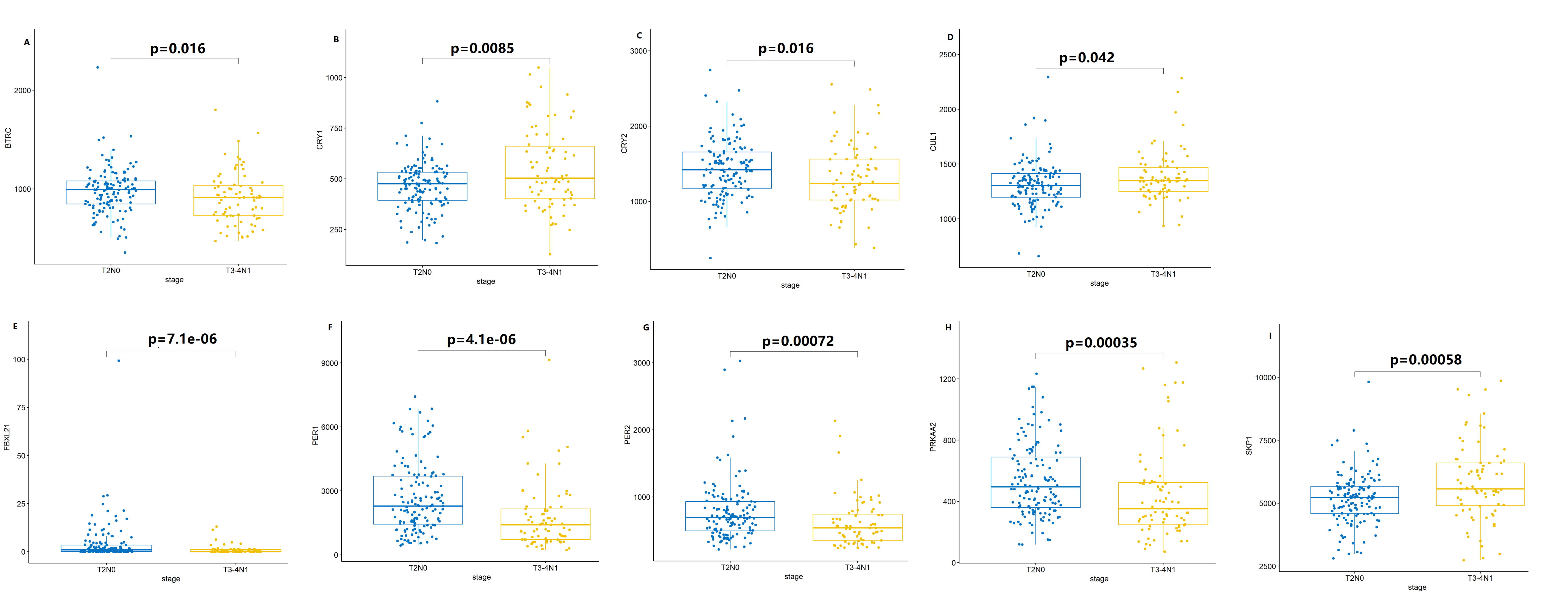

Supplement: Supplemental Information 1 [file peerj-09-12539-s001.png]

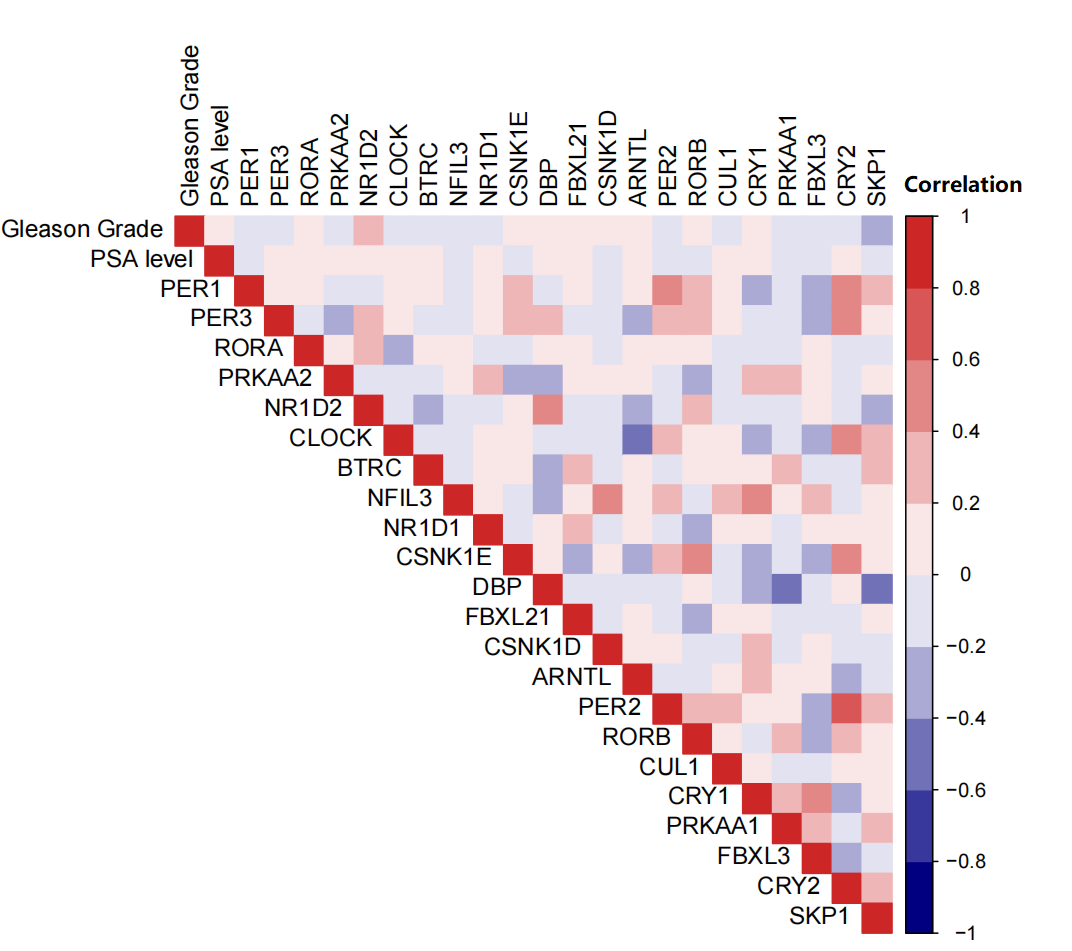

Supplement: Supplemental Information 2 [file peerj-09-12539-s002.png]

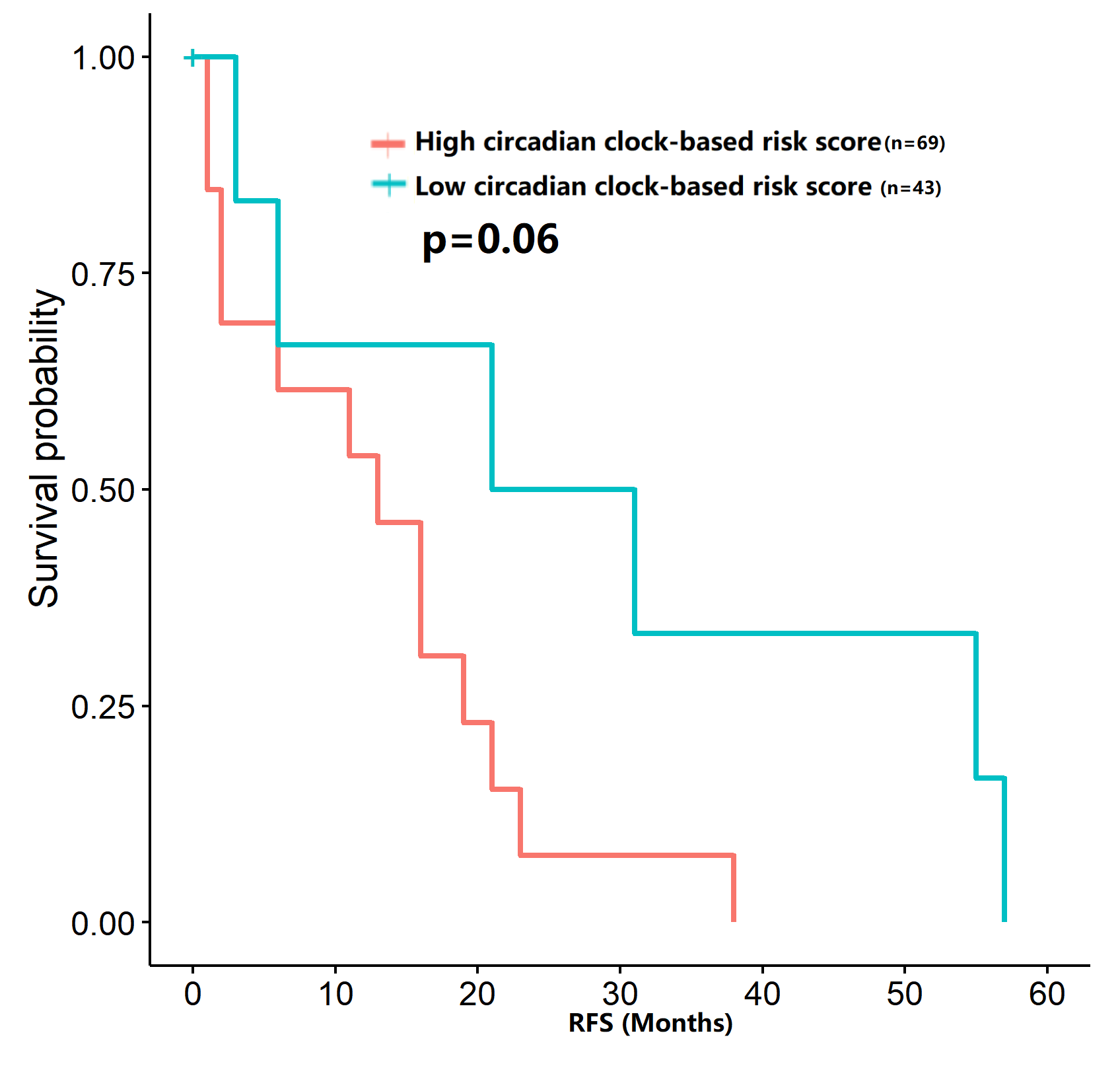

Supplement: Supplemental Information 3 — A total of 112 patients with prostate cancer obtained the recurrence free survival (RFS) data. [file peerj-09-12539-s003.png]

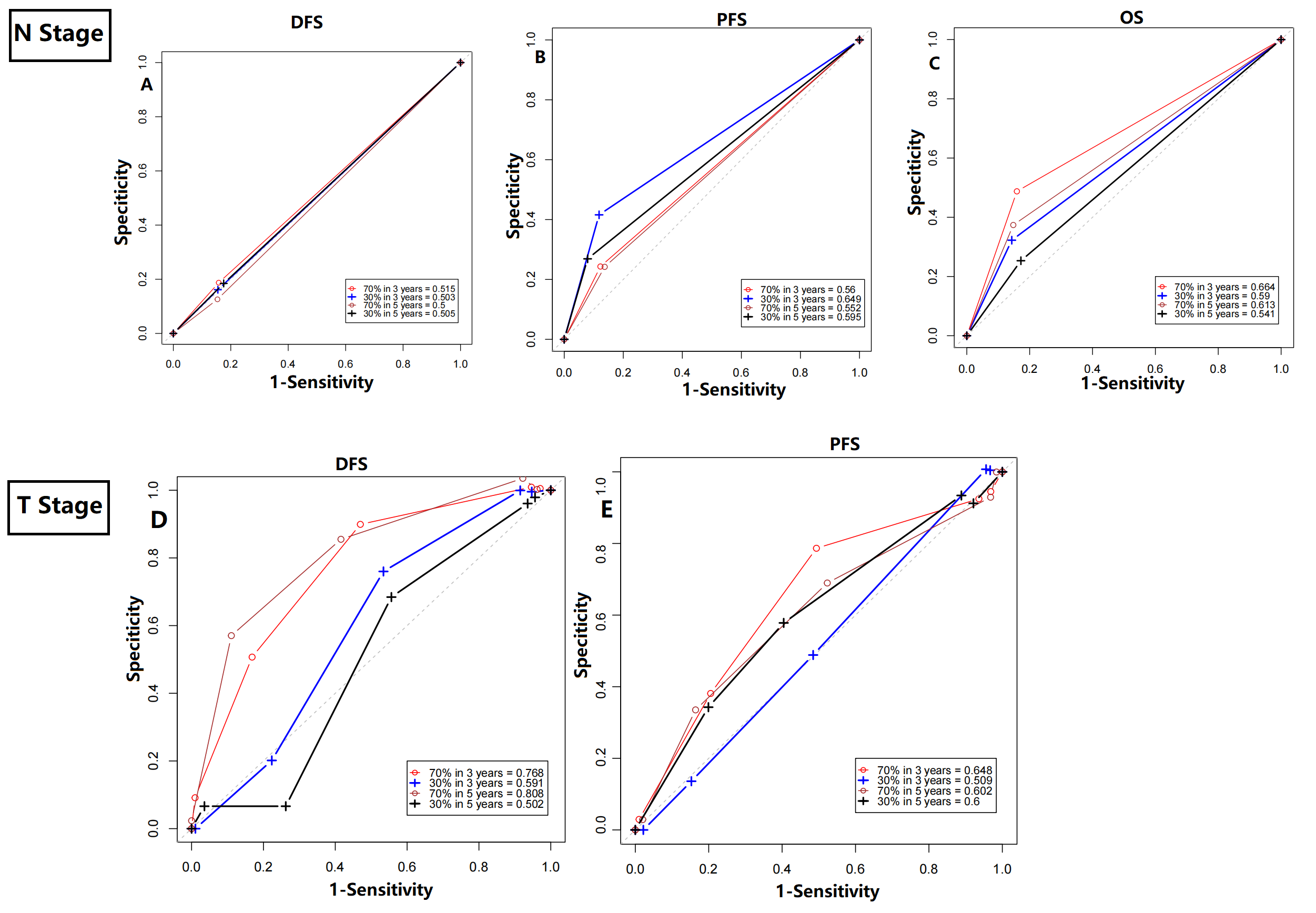

Supplement: Supplemental Information 4 — (A) For DFS, 3-year AUC values of N stage in the training cohort and the validation cohort were 0.515 and 0.503, respectively. The 5-year AUC values of the training cohort and the validation cohort were 0.500 and 0.505, respectively. (B) For PFS, 3-year AUC values of N stage in the training cohort and the validation cohort were 0.560 and 0.649, respectively. The 5-year AUC values of the training cohort and the validation cohort were 0.552 and 0.595, respectively. (C) For OS, 3-year AUC values of N stage in the training cohort and the validation cohort were 0.664 and 0.590, respectively. The 5-year AUC values of the training cohort and the validation cohort were 0.613 and 0.541, respectively. (D) For DFS, 3-year AUC values of T stage in the training cohort and the validation cohort were 0.768 and 0.591, respectively. The 5-year AUC values of the training cohort and the validation cohort were 0.808 and 0.502, respectively. (E) For PFS, 3-year AUC values of T stage in the training cohort and the validation cohort were 0.648 and 0.509, respectively. The 5-year AUC values of the training cohort and the validation cohort were 0.602 and 0.600, respectively. [file peerj-09-12539-s004.png]

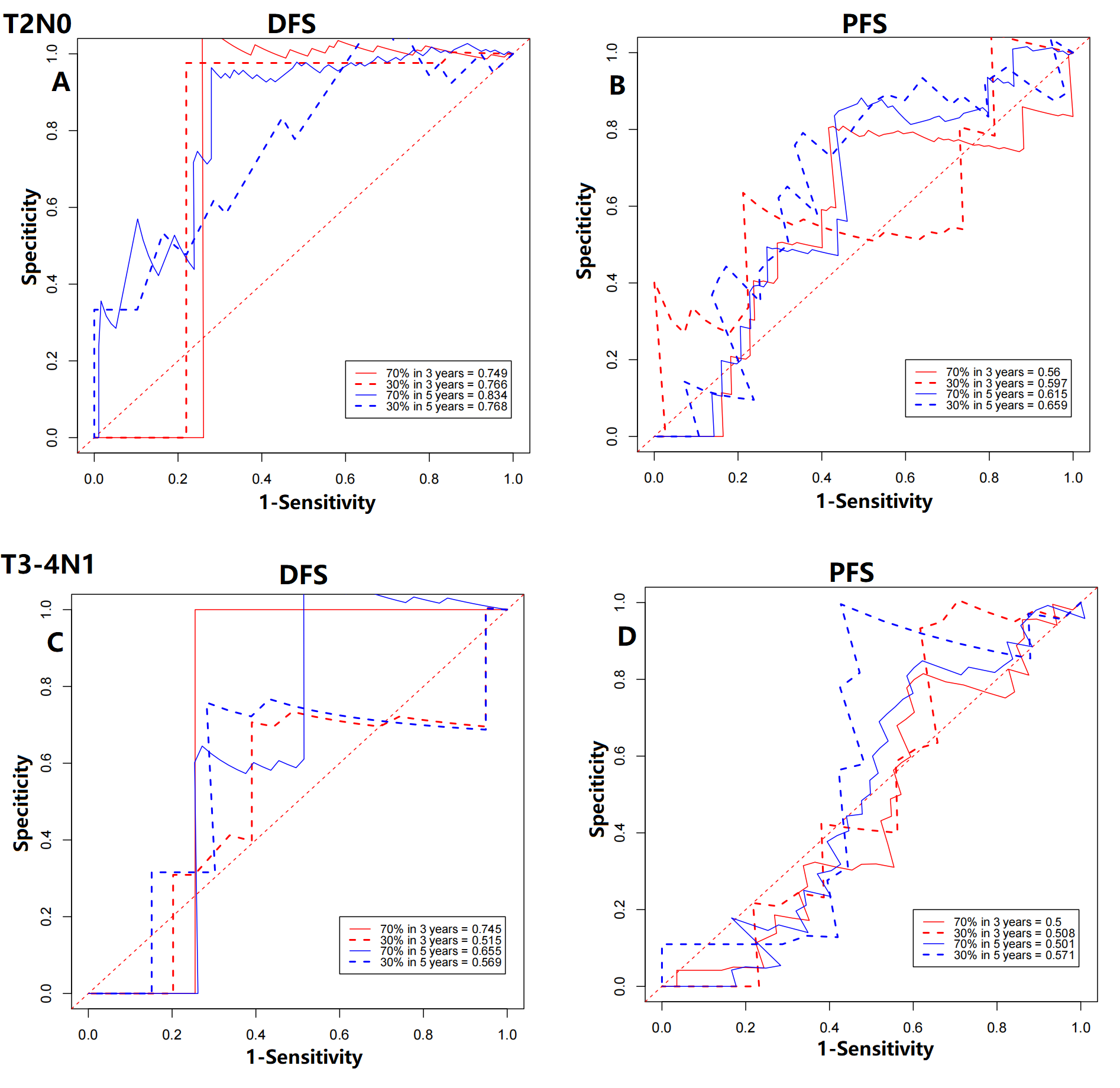

Supplement: Supplemental Information 5 — (A) For disease-free survival (DFS) at T2N0 stage, 3-year AUC values of the training cohort and the validation cohort were 0.749 and 0.766, respectively. The 5-year AUC values of the training cohort and the validation cohort were 0.834 and 0.768, respectively. (B) For progression-free survival (PFS) at T2N0 stage, 3-year AUC values of the training cohort and the validation cohort were 0.560 and 0.597, respectively. The 5-year AUC values of the training cohort and the validation cohort were 0.615 and 0.659, respectively. (C) For DFS at T3-4N1 stage, 3-year AUC values of the training cohort and the validation cohort were 0.745 and 0.515, respectively. The 5-year AUC values of the training cohort and the validation cohort were 0.665 and 0.569, respectively. (D) For PFS at T3-4N1 stage, 3-year AUC values of the training cohort and the validation cohort were 0.500 and 0.508, respectively. The 5-year AUC values of the training cohort and the validation cohort were 0.501 and 0.571, respectively. [file peerj-09-12539-s005.png]

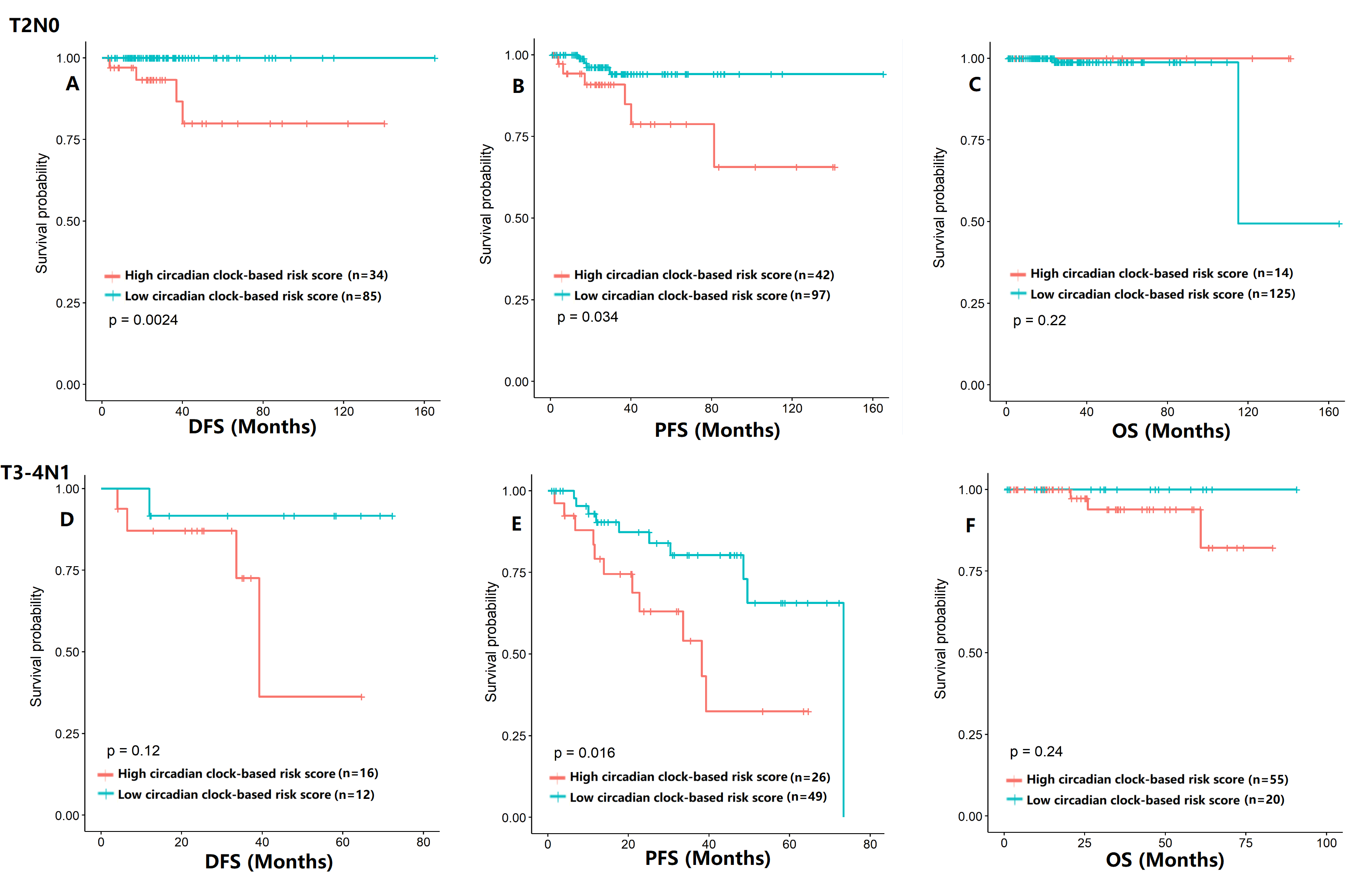

Supplement: Supplemental Information 6 — (A–C) Relationship between circadian clock-based risk score, disease-free survival (DFS; p = 0.0024), progression-free survival (PFS; p = 0.034), and overall survival (OS; p = 0.22) in T2N0 prostate cancer. D-F)Relationship between circadian clock-based risk score, DFS (p = 0.12), PFS (p = 0.016), and OS (p = 0.24) in T3-4N1 prostate cancer. [file peerj-09-12539-s006.png]

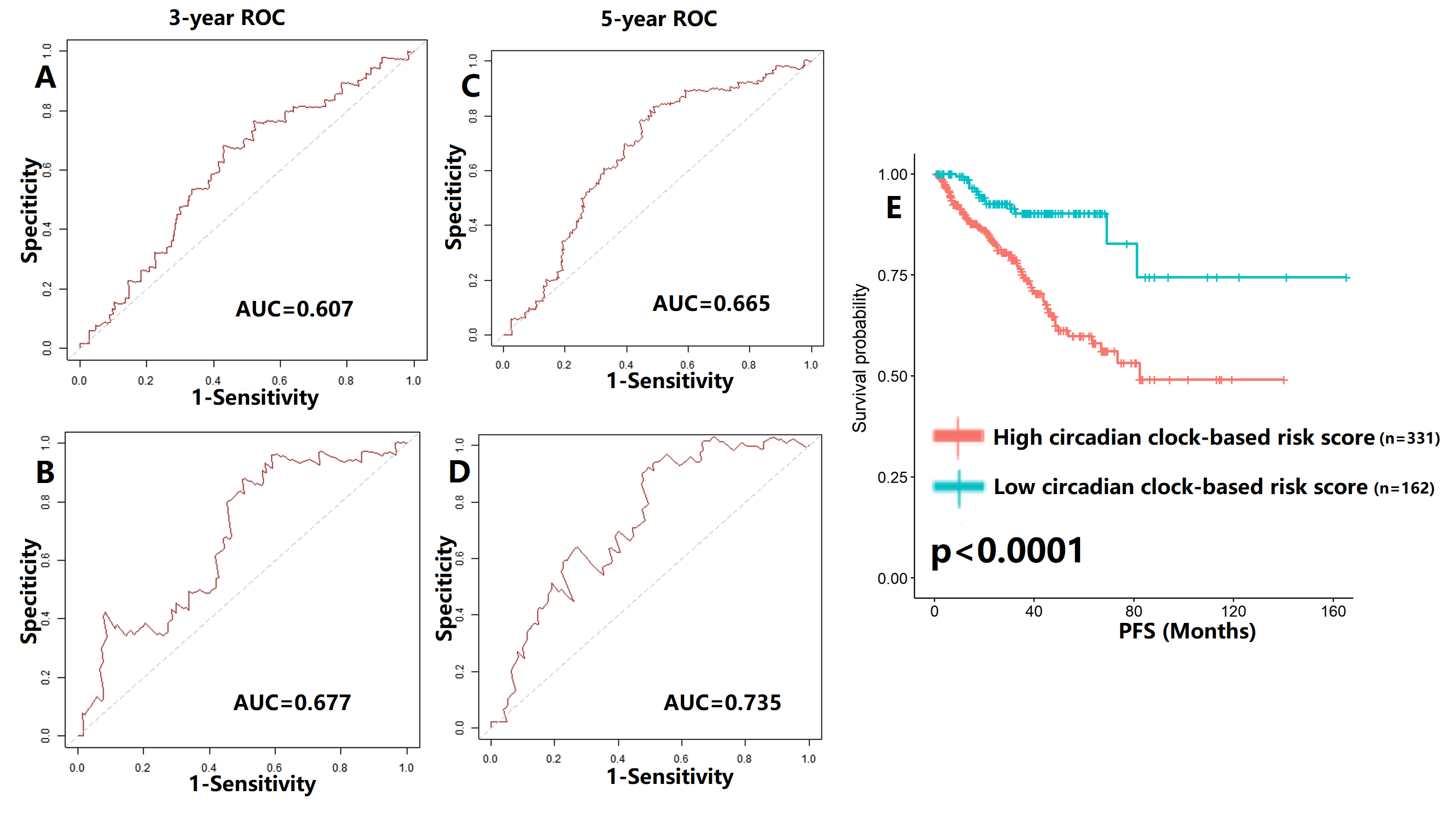

Supplement: Supplemental Information 7 — (A-B) ROC curves in the training cohort (AUC = 0.607) and the validation cohort (AUC = 0.677) for 3-year. (C-D) ROC curves in the training cohort (AUC = 0.665) and the validation cohort (AUC = 0.735) for 5-year. E) High circadian clock-based risk score was correlated with shorter PFS (p < 0.0001). [file peerj-09-12539-s007.png]

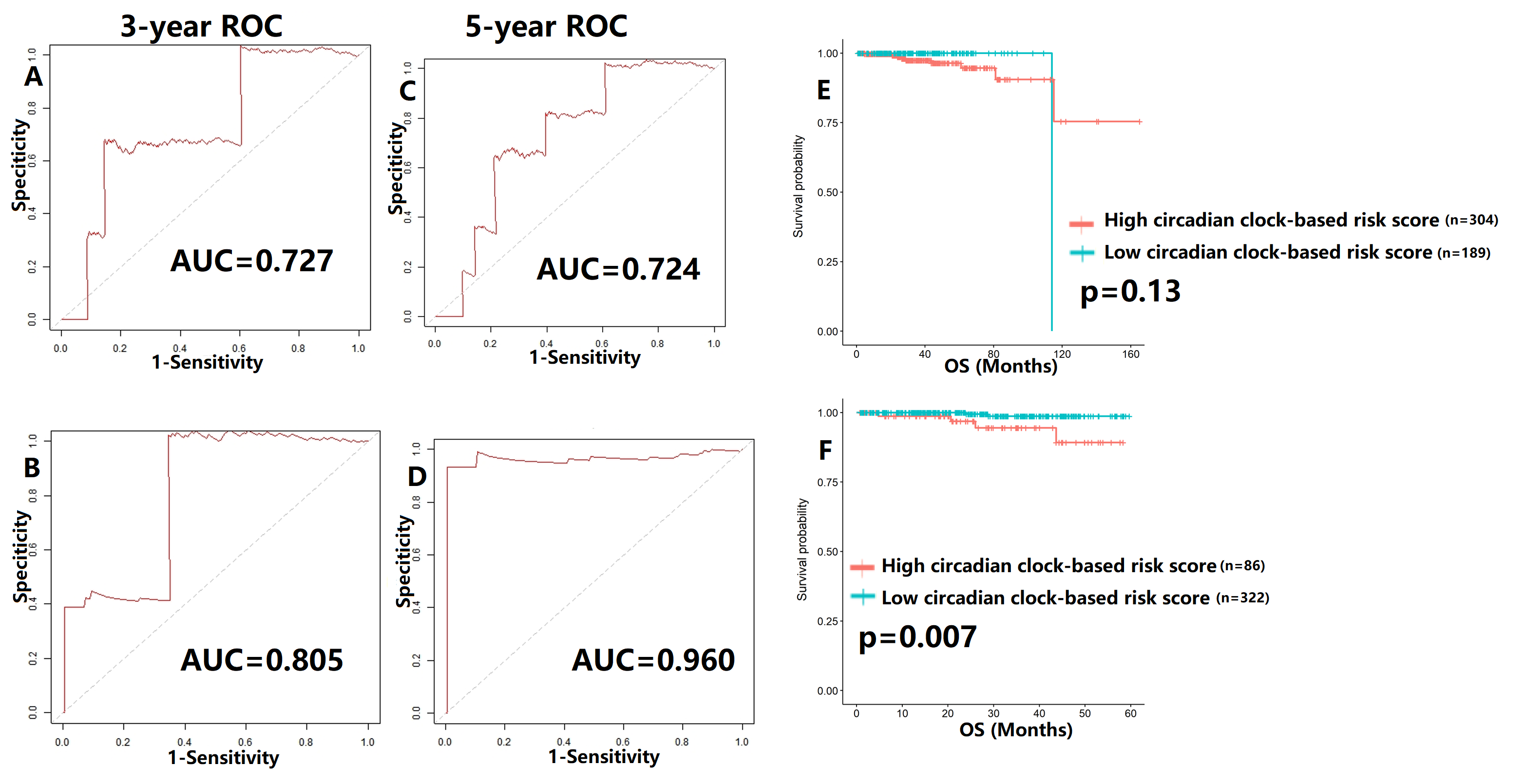

Supplement: Supplemental Information 8 — (A-B) ROC curves in the training cohort (AUC = 0.727) and the validation cohort (AUC = 0.805) for 3-year. (C-D) ROC curves in the training cohort (AUC = 0.724) and the validation cohort (AUC = 0.960) for 5-year. (E) The correlation between circadian clock-based risk score and OS (p = 0.13). (F) High circadian clock-based risk score was positively correlated with 5-year death rate (p = 0.007). [file peerj-09-12539-s008.png]

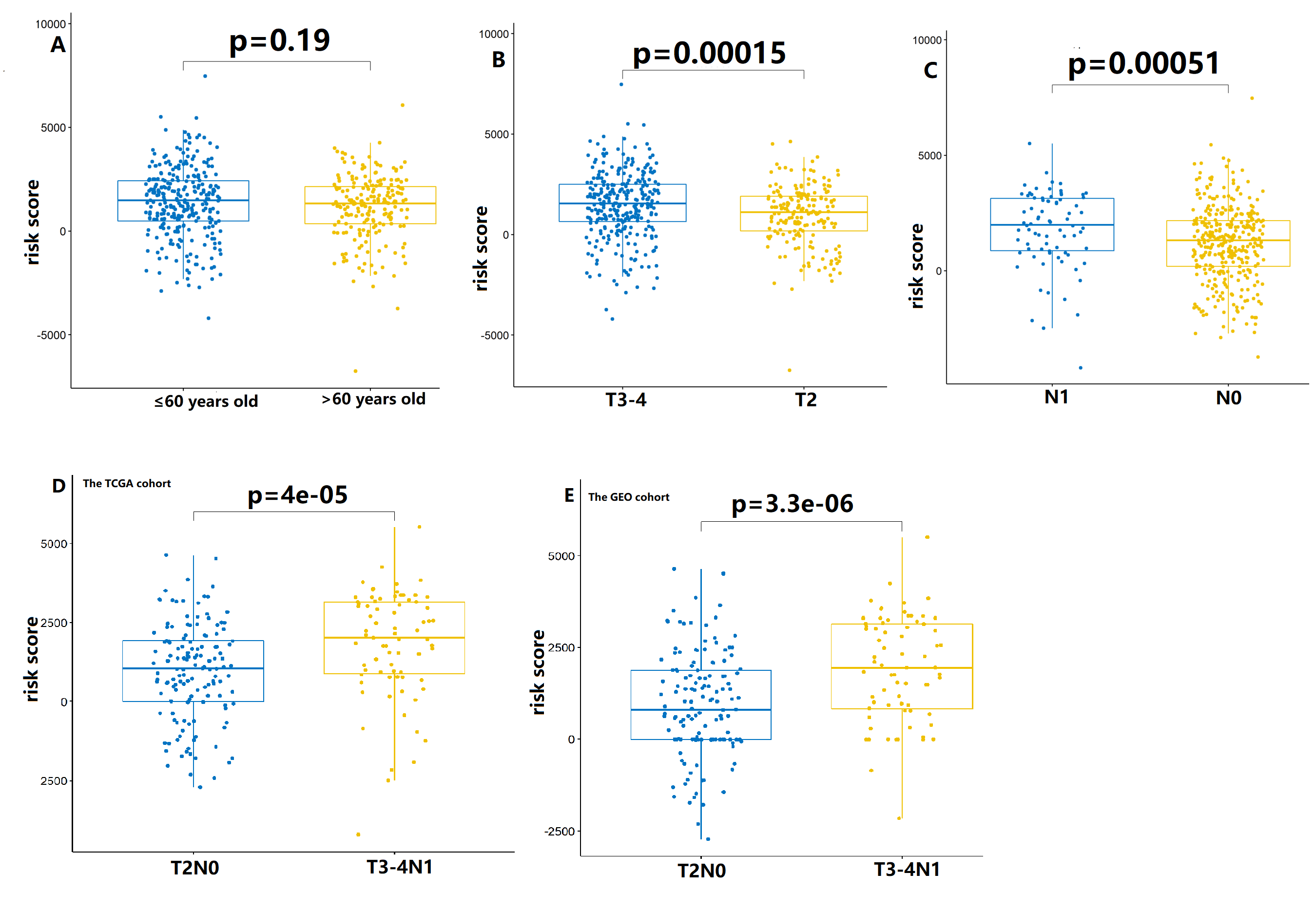

Supplement: Supplemental Information 9 [file peerj-09-12539-s009.png]

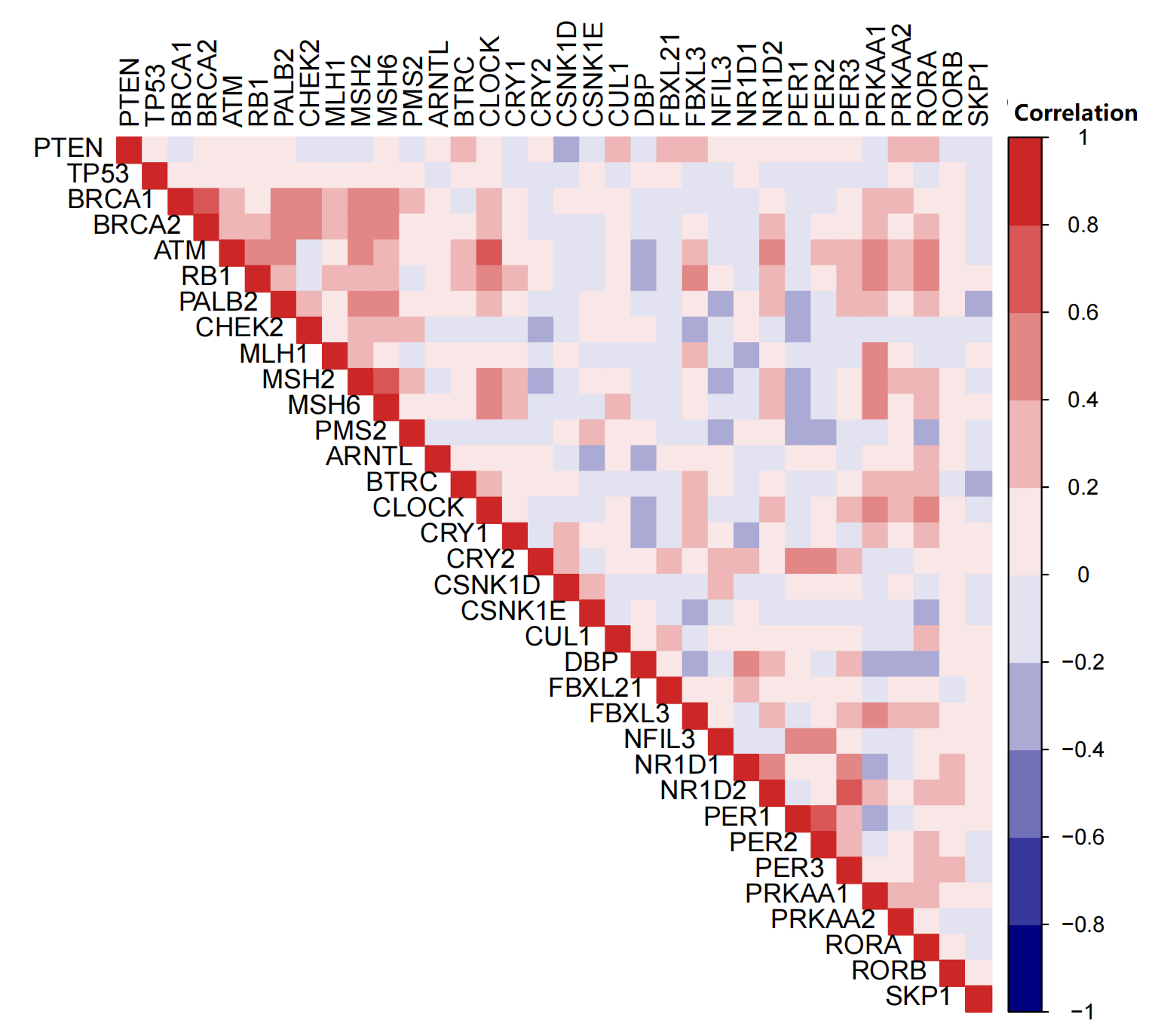

Supplement: Supplemental Information 10 [file peerj-09-12539-s010.png]
