# Supplementary material for: Prognostic values of the core components of the mammalian circadian clock in prostate cancer
Source: PeerJ. 2021 Dec 9;9:e12539. doi: 10.7717/peerj.12539 (PMC8667750; doi:10.7717/peerj.12539)
Supplement: Supplemental Information 11 [file peerj-09-12539-s011.docx]

**Table S1. Characteristics of the TCGA cohort and the GEO cohort**

| **Characteristics** | **The TCGA cohort (n=493)** | | **The GEO cohort(n=112)** | |
| --- | --- | --- | --- | --- |
|  | **Number, n** | **%** | **Number, n** | **%** |
| **Age, years** |  |  |  |  |
| <65 | 327 | 66.3% | 81 | 72.3% |
| ≧65 | 166 | 33.7% | 30 | 26.8% |
| NA | 0 | 0 | 1 | 0.9% |
| **Pathology T stage** |  |  |  |  |
| T1-2 | 186 | 37.7% | 34 | 30.4% |
| T3-4 | 300 | 60.9% | 77 | 68.7% |
| NA | 7 | 1.4% | 1 | 0.9% |
| **Pathology N stage** |  |  |  |  |
| N0 | 342 | 69.4% | 82 | 73.2% |
| N1 | 78 | 15.8% | 8 | 7.1% |
| NA | 73 | 14.8% | 22 | 19.6% |
| **Clinical T stage** |  |  |  |  |
| T1-2 | 0 | 0 | 95 | 84.8% |
| T3-4 | 0 | 0 | 16 | 14.3% |
| NA | 493 | 100% | 1 | 0.9% |
| **Clinical N stage** |  |  |  |  |
| N0 | 0 | 0 | 39 | 34.8% |
| N1 | 0 | 0 | 2 | 1.8% |
| NA | 493 | 100% | 71 | 63.4% |
| **[Neoadjuvant](C:/Users/%E9%99%88%E5%9F%B9%E6%AC%A3/AppData/Local/youdao/dict/Application/8.9.9.0/resultui/html/index.html#/javascript:;) [therapy](C:/Users/%E9%99%88%E5%9F%B9%E6%AC%A3/AppData/Local/youdao/dict/Application/8.9.9.0/resultui/html/index.html#/javascript:;)** |  |  |  |  |
| Yes | 2 | 0.4% | 0 | 0 |
| No | 491 | 99.6% | 0 | 0 |
| NA | 0 | 0 | 112 | 100% |
| **Surgery** |  |  |  |  |
| Yes | 493 | 100% | 112 | 100% |

Abbreviation: TCGA, the Cancer Genome Atlas; GEO, the Gene Expression Omnibus; N, lymph node; NA, not available; T, tumor.
