# Supplementary material for: Prognostic values of the core components of the mammalian circadian clock in prostate cancer
Source: PeerJ. 2021 Dec 9;9:e12539. doi: 10.7717/peerj.12539 (PMC8667750; doi:10.7717/peerj.12539)
Supplement: Supplemental Information 12 [file peerj-09-12539-s012.docx]

**Table S2. Relationship between disease-free survival (DFS) and expression levels of 22** **core components of the mammalian circadian clock (CCMCCs) in T2N0 prostate cancer (n=119).**

| **Gene** | **High expression group, n** | **Low expression group, n** | **Results** | **P value** |
| --- | --- | --- | --- | --- |
| ARNTL | 100 | 19 | High expression indicated longer DFS. | **0.035** |
| BTRC | 105 | 14 | High expression indicated longer DFS. | **0.038** |
| CLOCK | 108 | 11 | High expression indicated longer DFS. | **0.0013** |
| CRY1 | 108 | 11 | High expression indicated longer DFS. | **0.0087** |
| CRY2 | 92 | 27 | High expression indicated shorter DFS. | 0.23 |
| CSNK1D | 56 | 63 | High expression indicated shorter DFS. | **0.037** |
| CSNK1E | 49 | 70 | High expression indicated shorter DFS. | 0.17 |
| CUL1 | 12 | 107 | High expression indicated shorter DFS. | **0.0021** |
| DBP | 12 | 107 | High expression indicated shorter DFS. | **0.00013** |
| FBXL21 | 57 | 62 | High expression indicated longer DFS. | **0.04** |
| FBXL3 | 108 | 11 | High expression indicated longer DFS. | **0.0086** |
| NFIL3 | 81 | 38 | High expression indicated shorter DFS. | 0.14 |
| NR1D1 | 12 | 107 | High expression indicated shorter DFS. | **0.025** |
| NR1D2 | 94 | 25 | High expression indicated longer DFS. | 0.13 |
| PER1 | 103 | 16 | High expression indicated longer DFS. | 0.11 |
| PER2 | 108 | 11 | High expression indicated longer DFS. | **0.044** |
| PER3 | 99 | 20 | High expression indicated longer DFS. | **0.0027** |
| PRKAA1 | 102 | 17 | High expression indicated longer DFS. | **0.0011** |
| PRKAA2 | 108 | 11 | High expression indicated longer DFS. | **0.021** |
| RORA | 108 | 11 | High expression indicated longer DFS. | **0.0079** |
| RORB | 107 | 12 | High expression indicated longer DFS. | **0.0034** |
| SKP1 | 104 | 15 | High expression indicated longer DFS. | **0.045** |

Statistically significant data were marked with bold and underline.
