# Supplementary material for: Prognostic values of the core components of the mammalian circadian clock in prostate cancer
Source: PeerJ. 2021 Dec 9;9:e12539. doi: 10.7717/peerj.12539 (PMC8667750; doi:10.7717/peerj.12539)
Supplement: Supplemental Information 13 [file peerj-09-12539-s013.docx]

**Table S3. Relationship between disease-free survival (DFS) and expression levels of 22 core components of the mammalian circadian clock (CCMCCs) in T3-4N1 prostate cancer (n=75).**

| **Gene** | **High expression group, n** | **Low expression group, n** | **Results** | **P value** |
| --- | --- | --- | --- | --- |
| ARNTL | 11 | 64 | High expression indicated longer DFS. | 0.19 |
| BTRC | 44 | 31 | High expression indicated longer DFS. | **0.0077** |
| CLOCK | 68 | 7 | High expression indicated longer DFS. | **0.003** |
| CRY1 | 11 | 64 | High expression indicated shorter DFS. | **0.032** |
| CRY2 | 28 | 47 | High expression indicated shorter DFS. | 0.3 |
| CSNK1D | 39 | 36 | High expression indicated shorter DFS. | **0.048** |
| CSNK1E | 68 | 7 | High expression indicated shorter DFS. | 0.12 |
| CUL1 | 46 | 29 | High expression indicated shorter DFS. | 0.1 |
| DBP | 62 | 13 | High expression indicated longer DFS. | **0.0074** |
| FBXL21 | 20 | 55 | High expression indicated shorter DFS. | 0.077 |
| FBXL3 | 23 | 52 | High expression indicated longer DFS. | **0.039** |
| NFIL3 | 66 | 9 | High expression indicated shorter DFS. | 0.17 |
| NR1D1 | 68 | 7 | High expression indicated longer DFS. | **0.0059** |
| NR1D2 | 47 | 28 | High expression indicated longer DFS. | 0.084 |
| PER1 | 8 | 67 | High expression indicated shorter DFS. | 0.073 |
| PER2 | 30 | 45 | High expression indicated shorter DFS. | **0.03** |
| PER3 | 62 | 13 | High expression indicated longer DFS. | **0.048** |
| PRKAA1 | 47 | 28 | High expression indicated longer DFS. | 0.088 |
| PRKAA2 | 17 | 58 | High expression indicated longer DFS. | 0.13 |
| RORA | 60 | 15 | High expression indicated longer DFS. | **0.0087** |
| RORB | 25 | 50 | High expression indicated longer DFS. | 0.17 |
| SKP1 | 22 | 53 | High expression indicated shorter DFS. | **0.029** |

Statistically significant data were marked with bold and underline.
