# Supplementary material for: Prognostic values of the core components of the mammalian circadian clock in prostate cancer
Source: PeerJ. 2021 Dec 9;9:e12539. doi: 10.7717/peerj.12539 (PMC8667750; doi:10.7717/peerj.12539)
Supplement: Supplemental Information 14 [file peerj-09-12539-s014.docx]

**Table S4. Relationship between progression-free survival (PFS) and expression levels of 22 core components of the mammalian circadian clock (CCMCCs) in T2N0 prostate cancer (n=139).**

| **Gene** | **High expression group, n** | **Low expression group, n** | **Results** | **P value** |
| --- | --- | --- | --- | --- |
| ARNTL | 88 | 51 | High expression indicated longer PFS. | 0.12 |
| BTRC | 102 | 37 | High expression indicated longer PFS. | **0.026** |
| CLOCK | 60 | 79 | High expression indicated longer PFS. | **0.014** |
| CRY1 | 126 | 13 | High expression indicated longer PFS. | **0.037** |
| CRY2 | 124 | 15 | High expression indicated longer PFS. | 0.16 |
| CSNK1D | 79 | 60 | High expression indicated shorter PFS. | 0.32 |
| CSNK1E | 19 | 120 | High expression indicated longer PFS. | 0.15 |
| CUL1 | 16 | 123 | High expression indicated shorter PFS. | **0.0019** |
| DBP | 19 | 120 | High expression indicated shorter PFS. | **0.013** |
| FBXL3 | 126 | 13 | High expression indicated longer PFS. | **0.025** |
| FBXL21 | 59 | 80 | High expression indicated longer PFS. | **0.0034** |
| NFIL3 | 18 | 121 | High expression indicated shorter PFS. | 0.058 |
| NR1D1 | 14 | 125 | High expression indicated shorter PFS. | **0.0048** |
| NR1D2 | 21 | 118 | High expression indicated shorter PFS. | 0.056 |
| PER1 | 14 | 125 | High expression indicated shorter PFS. | **0.0071** |
| PER2 | 126 | 13 | High expression indicated longer PFS. | **0.01** |
| PER3 | 114 | 25 | High expression indicated longer PFS. | **0.00069** |
| PRKAA1 | 66 | 73 | High expression indicated longer PFS. | **0.047** |
| PRKAA2 | 126 | 13 | High expression indicated longer PFS. | 0.06 |
| RORA | 30 | 109 | High expression indicated shorter PFS. | 0.22 |
| RORB | 123 | 16 | High expression indicated longer PFS. | **<0.0001** |
| SKP1 | 123 | 16 | High expression indicated longer PFS. | 0.16 |

Statistically significant data were marked with bold and underline.
