# Supplementary material for: Prognostic values of the core components of the mammalian circadian clock in prostate cancer
Source: PeerJ. 2021 Dec 9;9:e12539. doi: 10.7717/peerj.12539 (PMC8667750; doi:10.7717/peerj.12539)
Supplement: Supplemental Information 15 [file peerj-09-12539-s015.docx]

**Table S5. Relationship between progression-free survival (PFS) and expression levels of 22 core components of the mammalian circadian clock (CCMCCs) in T3-4N1 prostate cancer (n=28).**

| **Gene** | **High expression group, n** | **Low expression group, n** | **Results** | **P value** |
| --- | --- | --- | --- | --- |
| ARNTL | 22 | 6 | High expression indicated shorter PFS. | 0.13 |
| BTRC | 26 | 2 | High expression indicated longer PFS. | **0.024** |
| CLOCK | 17 | 11 | High expression indicated shorter PFS. | 0.13 |
| CRY1 | 20 | 8 | High expression indicated longer PFS. | 0.3 |
| CRY2 | 12 | 16 | High expression indicated shorter PFS. | 0.088 |
| CSNK1D | 3 | 25 | High expression indicated shorter PFS. | 0.14 |
| CSNK1E | 4 | 24 | High expression indicated shorter PFS. | 0.2 |
| CUL1 | 6 | 22 | High expression indicated shorter PFS. | **0.012** |
| DBP | 24 | 4 | High expression indicated longer PFS. | **0.0016** |
| FBXL3 | 4 | 24 | High expression indicated longer PFS. | 0.19 |
| FBXL21 | 3 | 25 | High expression indicated shorter PFS. | 0.34 |
| NFIL3 | 4 | 24 | High expression indicated shorter PFS. | **0.0025** |
| NR1D1 | 24 | 4 | High expression indicated longer PFS. | **0.0016** |
| NR1D2 | 25 | 3 | High expression indicated longer PFS. | **0.0029** |
| PER1 | 23 | 5 | High expression indicated shorter PFS. | 0.28 |
| PER2 | 8 | 20 | High expression indicated shorter PFS. | 0.059 |
| PER3 | 26 | 2 | High expression indicated longer PFS. | **<0.0001** |
| PRKAA1 | 8 | 20 | High expression indicated longer PFS. | 0.099 |
| PRKAA2 | 23 | 5 | High expression indicated shorter PFS. | 0.26 |
| RORA | 12 | 16 | High expression indicated shorter PFS. | 0.089 |
| RORB | 19 | 9 | High expression indicated longer PFS. | **0.0056** |
| SKP1 | 23 | 5 | High expression indicated longer PFS. | 0.051 |

Statistically significant data were marked with bold and underline.
