# Supplementary material for: Prognostic values of the core components of the mammalian circadian clock in prostate cancer
Source: PeerJ. 2021 Dec 9;9:e12539. doi: 10.7717/peerj.12539 (PMC8667750; doi:10.7717/peerj.12539)
Supplement: Supplemental Information 16 [file peerj-09-12539-s016.docx]

**Table S6. Relationship between overall survival (OS) and expression levels of 22 core components of the mammalian circadian clock (CCMCCs) in T2N0 prostate cancer (n=139).**

| **Gene** | **High expression group, n** | **Low expression group, n** | **Results** | **P value** |
| --- | --- | --- | --- | --- |
| ARNTL | 22 | 117 | High expression indicated shorter OS. | **0.024** |
| BTRC | 60 | 79 | High expression indicated shorter OS. | **0.036** |
| CLOCK | 56 | 83 | High expression indicated longer OS. | 0.2 |
| CRY1 | 64 | 75 | High expression indicated shorter OS. | 0.13 |
| CRY2 | 71 | 68 | High expression indicated longer OS. | 0.2 |
| CSNK1D | 18 | 121 | High expression indicated longer OS. | 0.21 |
| CSNK1E | 14 | 125 | High expression indicated longer OS. | 0.22 |
| CUL1 | 126 | 13 | High expression indicated longer OS. | 0.14 |
| DBP | 41 | 98 | High expression indicated shorter OS. | 0.14 |
| FBXL21 | 57 | 82 | High expression indicated shorter OS. | 0.14 |
| FBXL3 | 103 | 36 | High expression indicated shorter OS. | 0.18 |
| NFIL3 | 100 | 39 | High expression indicated longer OS. | 0.1 |
| NR1D1 | 59 | 80 | High expression indicated longer OS. | **0.041** |
| NR1D2 | 116 | 23 | High expression indicated shorter OS. | 0.21 |
| PER1 | 126 | 13 | High expression indicated shorter OS | 0.37 |
| PER2 | 126 | 13 | High expression indicated longer OS. | 0.43 |
| PER3 | 75 | 64 | High expression indicated longer OS. | 0.095 |
| PRKAA1 | 56 | 83 | High expression indicated longer OS. | 0.21 |
| PRKAA2 | 92 | 47 | High expression indicated longer OS. | 0.054 |
| RORA | 70 | 69 | High expression indicated shorter OS | 0.13 |
| RORB | 22 | 117 | High expression indicated longer OS. | 0.076 |
| SKP1 | 115 | 24 | High expression indicated shorter OS. | 0.3 |

Statistically significant data were marked with bold and underline.
