# Supplementary material for: Prognostic values of the core components of the mammalian circadian clock in prostate cancer
Source: PeerJ. 2021 Dec 9;9:e12539. doi: 10.7717/peerj.12539 (PMC8667750; doi:10.7717/peerj.12539)
Supplement: Supplemental Information 17 [file peerj-09-12539-s017.docx]

**Table S7. Relationship between overall survival (OS) and expression levels of 22 core components of the mammalian circadian clock (CCMCCs) in T3-4N1 prostate cancer (n=75).**

| **Gene** | **High expression group, n** | **Low expression group, n** | **Results** | **P value** |
| --- | --- | --- | --- | --- |
| ARNTL | 36 | 39 | High expression indicated shorter OS. | **0.048** |
| BTRC | 34 | 41 | High expression indicated longer OS. | 0.13 |
| CLOCK | 47 | 28 | High expression indicated longer OS. | 0.12 |
| CRY1 | 49 | 26 | High expression indicated longer OS. | 0.18 |
| CRY2 | 59 | 16 | High expression indicated longer OS. | 0.14 |
| CSNK1D | 35 | 40 | High expression indicated shorter OS. | 0.1 |
| CSNK1E | 43 | 32 | High expression indicated longer OS. | **0.028** |
| CUL1 | 26 | 49 | High expression indicated shorter OS. | 0.15 |
| DBP | 63 | 12 | High expression indicated longer OS. | **0.027** |
| FBXL21 | 28 | 47 | High expression indicated longer OS. | 0.22 |
| FBXL3 | 48 | 27 | High expression indicated shorter OS. | 0.18 |
| NFIL3 | 28 | 47 | High expression indicated shorter OS. | 0.12 |
| NR1D1 | 26 | 49 | High expression indicated shorter OS. | 0.13 |
| NR1D2 | 31 | 44 | High expression indicated longer OS. | 0.14 |
| PER1 | 11 | 64 | High expression indicated shorter OS. | 0.28 |
| PER2 | 36 | 39 | High expression indicated shorter OS. | 0.056 |
| PER3 | 43 | 32 | High expression indicated shorter OS. | 0.15 |
| PRKAA1 | 15 | 60 | High expression indicated longer OS. | 0.26 |
| PRKAA2 | 40 | 35 | High expression indicated shorter OS. | 0.12 |
| RORA | 32 | 43 | High expression indicated longer OS. | 0.16 |
| RORB | 40 | 35 | High expression indicated longer OS. | 0.058 |
| SKP1 | 40 | 35 | High expression indicated longer OS. | 0.093 |

Statistically significant data were marked with bold and underline.
