# Supplementary material for: Prognostic values of the core components of the mammalian circadian clock in prostate cancer
Source: PeerJ. 2021 Dec 9;9:e12539. doi: 10.7717/peerj.12539 (PMC8667750; doi:10.7717/peerj.12539)
Supplement: Supplemental Information 18 [file peerj-09-12539-s018.docx]

**Table S8. Expression levels of CCMCCs between different mutation status of key prognostic genes in prostate cancer.**

|  |  | **ARNTL** | **BTRC** | **CLOCK** | **CRY1** | **CRY2** | **CSNK1D** | **CSNK1E** | **CUL1** | **DBP** | **FBXL3** | **NFIL3** | **NR1D1** | **NR1D2** | **PER1** | **PER2** | **PER3** | **PRKAA1** | **PRKAA2** | **RORA** | **RORB** | **SKP1** |
| --- | --- | --- | --- | --- | --- | --- | --- | --- | --- | --- | --- | --- | --- | --- | --- | --- | --- | --- | --- | --- | --- | --- |
| **PTEN** | **P** | 0.192 | **0.000000515** | **0.0102** | 0.286 | **0.0179** | 0.92 | 0.935 | **0.0003074** | 0.468 | **0.0231** | **0.00002905** | 0.33 | **0.0008006** | **0.00004203** | **0.00001296** | 0.152 | **0.00005925** | **0.000006563** | 0.217 | **0.00000054** | 0.331 |
|  | **HEP** | WT | WT | M | M | WT | WT | M | WT | M | WT | WT | M | M | WT | WT | M | M | WT | WT | M | M |
| **TP53** | **P** | 0.228 | **0.001362** | 0.934 | **0.003774** | 0.92 | 0.0659 | **0.0004053** | 0.203 | 0.106 | 0.173 | **0.0393** | 0.286 | 0.0523 | **0.0003505** | 0.0805 | 0.199 | 0.418 | 0.205 | 0.314 | **0.006969** | 0.11 |
|  | **HEP** | WT | WT | M | M | WT | M | M | M | M | WT | WT | M | M | WT | WT | M | M | WT | WT | M | M |
| **BRCA1** | **P** | 0.923 | 0.93 | 0.946 | 0.92 | 0.563 | 0.332 | 0.867 | 0.953 | 0.418 | 0.613 | 0.324 | 0.252 | 0.444 | 0.199 | 0.125 | 0.739 | 0.523 | 0.524 | 0.225 | 0.249 | 0.664 |
|  | **HEP** | M | WT | M | M | WT | M | M | M | M | M | M | WT | WT | WT | WT | WT | M | WT | M | WT | M |
| **BRCA2** | **P** | 0.324 | 0.192 | 0.82 | 0.517 | 0.0851 | 0.965 | 0.0909 | 0.979 | 0.459 | 0.316 | 0.952 | 0.17 | 0.982 | 0.64 | 0.309 | 0.883 | 0.561 | 0.521 | 0.934 | 0.519 | 0.939 |
|  | **HEP** | M | WT | WT | WT | WT | M | WT | WT | M | WT | WT | M | WT | M | WT | M | WT | M | WT | M | M |
| **ATM** | **P** | 0.768 | 0.481 | **0.009059** | 0.564 | 0.936 | 0.24 | 0.168 | 0.248 | 0.369 | 0.347 | 0.264 | 0.917 | 0.288 | 0.43 | 0.76 | 0.78 | 0.668 | 0.436 | 0.0199 | 0.838 | 0.366 |
|  | **HEP** | M | M | M | M | WT | WT | WT | M | M | WT | WT | M | M | M | M | M | WT | M | M | M | WT |
| **RB1** | **P** | 0.451 | 0.966 | 0.87 | 0.0441 | 0.001714 | 0.15 | 0.854 | 0.963 | 0.214 | 0.281 | 0.0291 | 0.754 | 0.559 | 0.132 | 0.0594 | 0.0415 | 0.437 | 0.238 | 0.0864 | 0.252 | 0.26 |
|  | **HEP** | M | WT | WT | WT | WT | WT | M | WT | M | WT | WT | WT | WT | WT | WT | WT | WT | M | WT | M | WT |
| **PALB2** | **P** | **0.048** | 0.527 | 0.0679 | 0.111 | 0.764 | 0.338 | 0.513 | 0.876 | 0.0451 | 0.964 | 0.574 | 0.26 | 0.284 | 0.256 | 0.111 | 0.131 | 0.439 | 0.0856 | 0.885 | 0.416 | 0.569 |
|  | **HEP** | M | WT | M | M | M | M | M | WT | WT | WT | M | WT | M | M | M | M | M | M | M | M | WT |
| **CHEK2** | **P** | 0.992 | 0.001034 | 0.431 | 0.548 | 0.0992 | 0.233 | 0.21 | 0.091 | 0.624 | 0.0683 | 0.63 | 0.416 | 0.111 | 0.165 | 0.382 | 0.134 | 0.123 | 0.466 | 0.261 | 0.113 | 0.903 |
|  | **HEP** | WT | M | M | M | M | M | M | WT | M | M | WT | M | M | WT | M | M | M | WT | M | M | M |
| **MLH1** | **P** | 0.607 | 0.698 | 0.571 | 0.595 | 0.23 | 0.3 | 0.516 | 0.129 | 0.653 | 0.881 | 0.215 | 0.946 | 0.933 | 0.322 | 0.591 | 0.884 | 0.213 | 0.618 | 0.198 | 0.208 | 0.682 |
|  | **HEP** | WT | WT | WT | M | WT | M | M | M | WT | WT | WT | M | WT | WT | WT | M | M | M | WT | WT | WT |
| **MSH2** | **P** | 0.152 | 0.332 | 0.49 | 0.168 | 0.882 | 0.744 | 0.437 | 0.0177 | 0.0003112 | 0.776 | 0.98 | 0.902 | 0.88 | 0.0554 | 0.482 | 0.457 | 0.939 | 0.265 | 0.244 | 0.538 | 0.696 |
|  | **HEP** | M | M | WT | WT | M | M | M | WT | M | WT | WT | WT | WT | WT | WT | WT | WT | M | WT | WT | WT |
| **MSH6** | **P** | 0.247 | 0.987 | 0.768 | 0.806 | 0.125 | 0.577 | 0.84 | 0.863 | 0.299 | 0.497 | 0.691 | 0.275 | 0.295 | 0.454 | 0.244 | 0.244 | 0.803 | 0.249 | 0.293 | 0.0467 | 0.844 |
|  | **HEP** | M | M | WT | WT | WT | M | WT | WT | WT | WT | WT | WT | WT | WT | WT | WT | WT | M | WT | WT | M |
| **PMS2** | **P** | 0.777 | 0.849 | 0.885 | 0.46 | 0.229 | 0.407 | 0.687 | 0.598 | 0.502 | 0.465 | 0.297 | 0.652 | 0.921 | 0.0228 | 0.206 | 0.387 | 0.599 | 0.672 | 0.443 | 0.221 | 0.465 |
|  | **HEP** | M | WT | WT | WT | WT | M | WT | M | M | WT | WT | WT | WT | WT | WT | WT | WT | M | WT | WT | M |

Abbreviation: CCMCCs, core components of the mammalian circadian clock; HEP, high expression group; WT, wild type group; M, mutation group.
